# Genetic link between primary biliary cholangitis and connective tissue diseases in European populations: A two-sample Mendelian randomization study

**DOI:** 10.1371/journal.pone.0298225

**Published:** 2024-02-09

**Authors:** Zhekang Liu, Yijia Shao, Xinwang Duan

**Affiliations:** Rheumatology and Immunology Department, The Second Affiliated Hospital of Nanchang University, Nanchang, Jiangxi, China; University Hospital of Bologna Sant’Orsola-Malpighi Polyclinic Department of Digestive System: Azienda Ospedaliero-Universitaria di Bologna Policlinico Sant’Orsola-Malpighi Dipartimento dell’apparato digerente, ITALY

## Abstract

**Background:**

An association between primary biliary cholangitis (PBC) and connective tissue diseases (CTDs) [rheumatoid arthritis (RA), systemic lupus erythematosus (SLE), Sjögren’s syndrome (SS), systemic sclerosis (SSc)] has been found in observational studies. However, the direction causality is unclear. The aim of this study was to assess the causality between PBC and CTDs and to promote early screening, pre-emptive therapy, and accurate stratification.

**Methods:**

A two-sample Mendelian randomization (MR) analysis was performed to assess the causal relationship between PBC [Genome-Wide Association Study (GWAS) meta-analysis, 8021 cases/16498 controls], and SLE (GWAS meta-analysis, 8021 cases/16489 controls), RA(FinnGen, 6236 cases/14727 controls), SS(FinnGen, 2495 cases/365533 controls), SSc (FinnGen, 302 cases/213145 controls). Inverse variance weighting (IVW) was used as the primary analysis method, supplemented by four sensitivity analyses to assess the robustness of the results.

**Results:**

The IVW revealed that genetically predicted PBC increased the risk of SLE [odd’s ratio (OR) = 1.43, 95% confidence interval (CI) 1.30–1.58, P < 0.001]), RA (OR = 1.09, 95%CI1.04–1.14, P<0.001), and SS (OR = 1.18, 95%CI1.12–1.24, P<0.001), but not that of SSc. In addition, no association was observed between CTDs as an exposure and PBC. Sensitivity analyses did not reveal horizontal pleiotropy.

**Conclusions:**

Our study provided new genetic evidence for a causal relationship between PBC and CTDs. PBC increased the risk of SLE, RA, and SS. Our findings highlighted the importance of active screening and intervention for CTDs in patients with PBC.

## 1 Introduction

Primary biliary cholangitis(PBC) is a chronic, inflammatory, immune-mediated liver injury [1]. Until 2016, it was called primary cirrhosis [2, 3]. PBC is a multi-causal disease in which the immune system triggers an attack by targeting and destroying epithelial cells of the bile duct [3]. Although the liver is the main target organ of PBC, PBC may affect multiple systems, such as arthritis [4], interstitial lung disease [5, 6], pulmonary hypertension [7] and nephritis [8]. These coexisting symptoms often make the diagnosis and treatment more difficult, and can even alter the course, prognosis and outcome of PBC.

PBC is distinguished from connective tissue diseases(CTDs). However, in observational studies, a combination of CTDs was observed in patients with chronic biliary cholangitis, with the most frequently reported CTDs as Sjögren’s syndrome(SS), A recent meta-analysis reported that 35% [(95% confidence interval (CI): 28%–-42%]) of patients with PBC had combined SS [9], This may be related to the fact that PBC and Sjogren’s syndrome share common epithelial cell immune changes in epithelial cells. Certainly, A previous study that report the clinical features of 34 patients with PBC combined with systemic lupus erythematosus (SLE) and distinguish liver injury in SLE from PBC [10]. In addition, one study noted that studies of extrahepatic manifestations of PBC have focused on rheumatic diseases and reported the prevalence of various rheumatic diseases in 361 patients [11]. In addition to the overlap between the diseases, it noted that CTDs such as SLE, systemic sclerosis (SSC), SS, and inflammatory myopathy (IM)have a higher rate of positivity for the AMA signature antibody (anti-mitochondrial antibody) than the normal population [12]. A large number of clinical studies are available on PBC and CTDs, and some research has been done on animal models and immune mechanisms of PBC [13], However, this is not matched by genetic epidemiological studies of PBC and extrahepatic CTDs. In addition, early identification of autoimmune diseases associated with PBC is recommended in the guidelines for its management [1], Therefore, genetic epidemiologic studies of PBC and extrahepatic CTDs are necessary. Exploring the relationship between PBC and extrahepatic CTDs can help to enhance the understanding of the pathogenesis of PBC and CTDs and to develop appropriate therapeutic approaches.

Although traditional case-control or cross-sectional studies have proposed a relationship between PBC and CTDs, most are observational designs with small sample sizes [14, 15], therefore, they exhibit the limitations of lack of valid inferences of effects, and susceptibility to multiple confounding factors. At the same time, due to the lower prevalence of PBC, there is a lack of cohort studies with larger numbers of patients. With the continued development of new statistical methods, genome-wide association study (GWAS) data, epigenetics and various genomics, Mendelian randomization (MR) studies are more widely used to explore causal relationships between complex exposures and disease. Three basic assumptions need to be met when performing MR analyses: genetic variation is 1. associated with exposure, 2. not associated with confounders, and 3. not associated with outcome. The rationale of MR lies in equal, random and independent genetic variation during meiosis and uses genetic variation as an instrumental variable. The properties of MR minimize confounding factors and reverse causality, and it is second only to randomized control trials (RCTs) in terms of level of evidence. Therefore, in this study, the correlation between PBC and various CTDs was assessed by two-sample MR [16]. This study provides provided insights early screening, early treatment, and disease stratification in patients with PBC.

## 2 Methods

### 2.1 Two-sample MR

**Fig 1:**
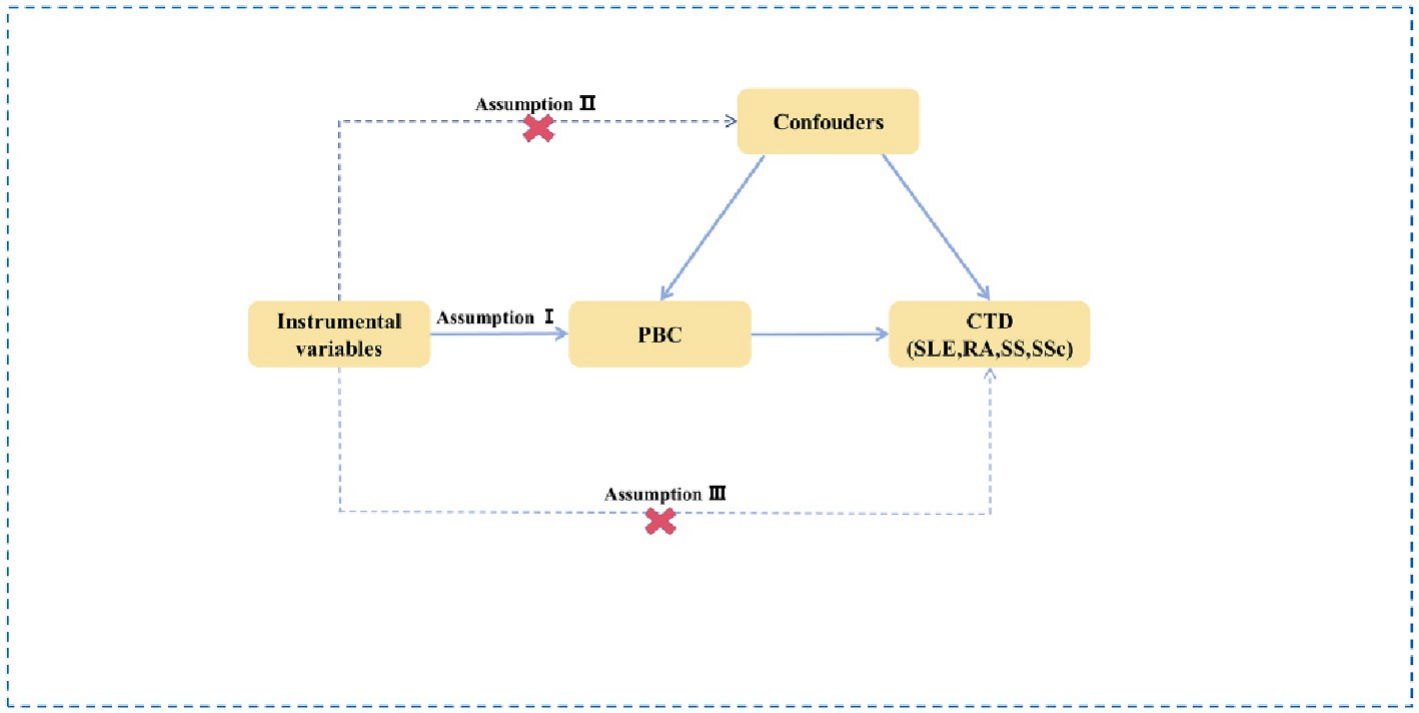
Diagram of the two-sample Mendelian randomization (MR) study for the association between PBC and CTDs. PBC, primary biliary cholangitis; SLE, systemic lupus erythematosus; RA, rheumatoid arthritis; SS, Sjögren’s syndrome; SSc, systemic sclerosis.

A two-sample MR Study was conducted to assess whether there is a causal relationship between the genetic susceptibility of PBC and CTDs (including RA, SLE, SS, SSc). Multiple single nucleotide polymorphisms (SNPs) were defined as instrumental variables (IVs). The three key assumptions of the MR Analysis are as follows (Fig 1): 1. IVs are strongly related to exposure factors; 2. IVs are independent of any confounding factors; and 3. The selected IVs is not directly related to the outcome variable.

### 2.2 Data sources

The GWAS data is a genome-wide association study of the largest European population and provides the basis for use in studies of PBC, SLE, RA, and SSc (https://www.ebi.ac.uk/gwas/). Data for the PBC were derived from a meta-analysis of GWAS, and the data were derived from the cohorts of three samples: 1. the Canada-UK cohort included 4,615 cases and 9,213 controls, 2. the Italian cohort included 255 cases and 579 controls, and 3. the U.S. cohort including 891 cases and 621 controls. The summary statistics for SLE (5,201 patients and 9066 controls) was obtained from the GWAS catalog(https://www.ebi.ac.uk/gwas/). Summary-level statistical data for RA, SS, SSc were obtained from the FinnGen GWAS results. The GWAS data used in this study is given in Table 1.

**Table 1:** Genome-wide association study (GWAS) datasets used in the study.

Phenotypes	Source	Phenotypic code	Cases/controls	Ancestry
PBC	GWAS meta-analysis [39]	GCST90061440	8021/16489	European
SLE	US, Italy, Netherlands, Turkey, Spain [40]	GCST003156	5201/9066	European
RA	UK, Finland	ebi-a-GCST90018910	8255/409001	European
SS	FinnGen	R9_M13_SJOGREN	2495/365533	European
SSc	UK	GCST90044535	104/456,244	European

### 2.3 Selection of IVs

For genetical instruments of PBC and CTDs, we used single nucleotide polymorphisms (SNPs) that were strongly associated with each exposure (p < 5 × 10−8) as instruments. We screened LD independent SNPs based on R2 = 0.001, window size = 10,000 kb. Phenotypes associated with each genetic variant were examined using the PhenoScanner software. SNPs overlapped with the risk of outcome were eliminated to prevent potential multiple effects. And “harmonise_data” code function was used to remove the palindromic SNPs in the MR analysis.

### 2.4 Statistical analyses

The “TwoSampleMR” packages of the R software (version 4.2.2) were used to perform MR analysis. Several MR methods were used to infer causal relationships between PBC and CTDs(SLE, RA, SS, and SSc). These methods included inverse variance weighting (IVW), MR-Egger, median weighting, and Weight mode. IVW method is regarded as the most important method in the study. To assess the association between PBC and the four CTDs, MR Analysis was performed. IVW was considered to be the primary MR method, and p < 0.05 and consistent direction of the four MR methods were considered significant. When the heterogeneity test shows heterogeneity, multiplicative random effects will be used, otherwise, a fixed effects model will be used.

### 2.5 Sensitivity analyses

We performed “leave-one-out” sensitivity analyses to explore the possibility that causality is driven by a single SNP. We used Cochran’s Q statistic to estimate heterogeneity, in which case we used the random effects IVW test to provide a more conservative and robust estimates.

## 3. Results

### 3.1. IVs

SNPs associated with these diseases were selected as IVs based on the established quality control criteria. Each of these SNPS was associated with exposure and not with outcome. The F-statistics of these SNPs were above the threshold of 10, indicating that they strongly represented these diseases in the MR analysis.

### 3.2. Impact of PBC on CTD

**Fig 2:**
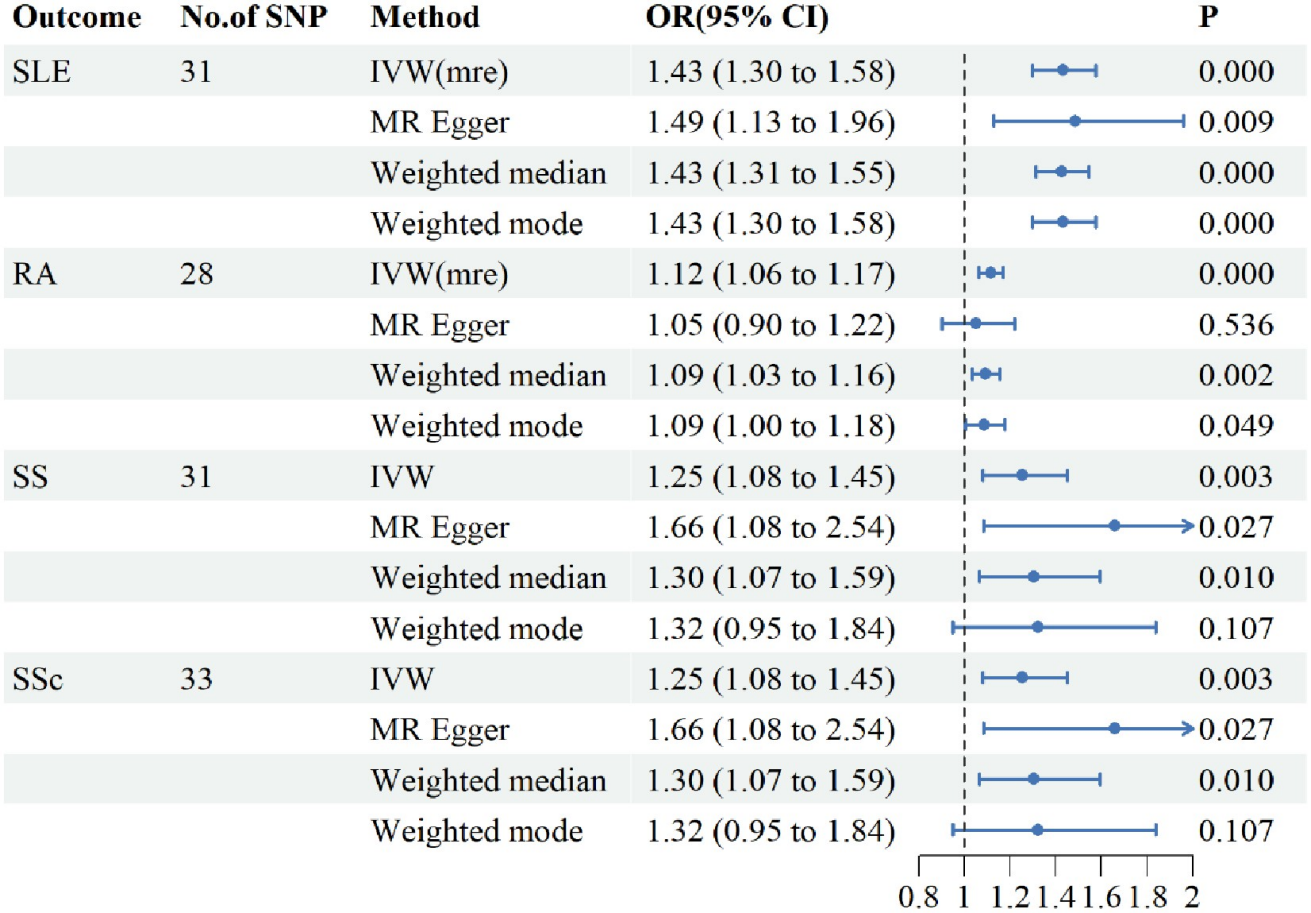
Causal effects of PBC on the risk of CTDs assessed by different Mendelian randomization methods. PBC for exposure, CTDs for outcome. PBC, primary biliary cholangitis; SLE, systemic lupus erythematosus; RA, rheumatoid arthritis; SS, Sjögren’s syndrome; SSc, systemic sclerosis. SNP, single-nucleotide polymorphism; OR, odds ratio; CI, confidence interval. IVW, inverse-variance weighted; IVW(mre), inverse-variance weighted(multiplicative random effects).

Genetic variants of PBC were associated with SLE, RA, and SS (Fig 2). According to IVW method, the presence of PBC increased the risk of developing SLE by 43% (OR = 1.43, 95%CI 1.30–1.58, P<0.001), increased the risk of RA by 12% (OR = 1.12, 95%CI1.06–1.17, P<0.001), as well as increased the risk of SS by 18% (OR = 1.18, 95% CI1.12–1.24, P<0.001) and increased the risk of SSc by 25% (OR = 1.25, 95% CI1.08–1.45, P<0.001). As heterogeneity was detected during the analysis of the RA and SSc database, we used the IVW method(multiplicative random effects). Meanwhile, a leave-one-out method was performed to verify the robustness of the MR analysis and ensure the reliability of the results. MR-egger revealed no significant horizontal pleiotropy between PBC and CTDs, and the results can be seen in Table 2. All included SNPs were tested for heterogeneity, and a random effects model was used if heterogeneity existed (Table 2).

**Table 2:** Heterogeneity and pleiotropy tests for the associations between PBC and CTD.

MR analysis	Heterogeneity test		Pleiotropy test		
	Q	Q-pval	Egger_intercept	se	p
PBC-SLE	111.110	<0.001	-0.009	0.032	0.776
PBC-RA	125.150	<0.001	0.006	0.015	0.708
PBC-SS	27.007	0.571	0.001	0.019	0.941
PBC-SSc	34.035	0.653	0.092	0.073	0.216
SLE-PBC	46.706	<0.001	0.0509	0.034	0.162
RA-PBC	21.723	0.001	-0.041	0.050	0.442
SS-PBC	3.188	0.074	NA	NA	NA
SSc-PBC	0.869	0.833	-0.068	0.044	0.219

### 3.3. Impact of CTD on PBC

**Fig 3:**
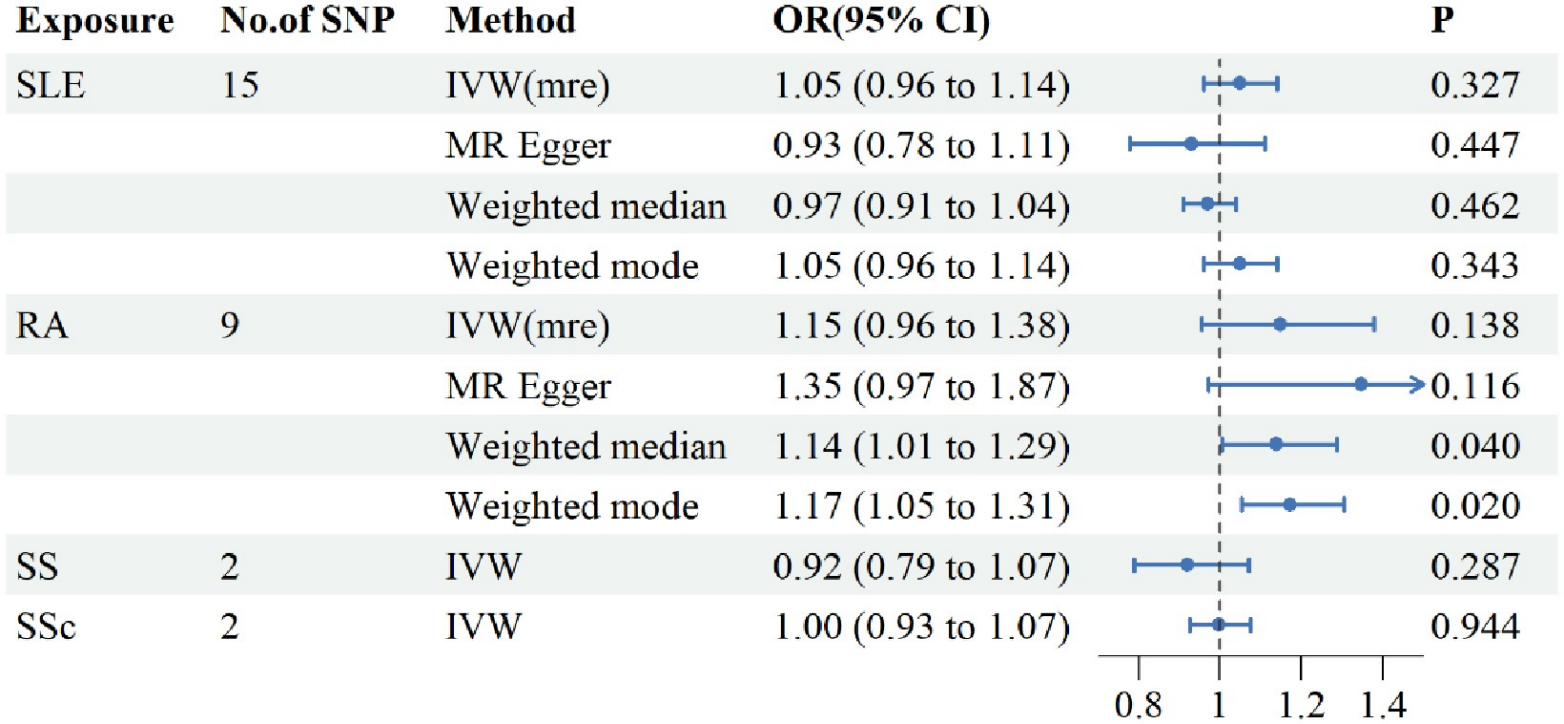
Causal effects of CTDs on the risk of PBC assessed by inverse Mendelian randomization method. CTDs for exposure, PBC for outcome. PBC, primary biliary cholangitis; SLE, systemic lupus erythematosus; RA, rheumatoid arthritis; SS, Sjögren’s syndrome; SSc, systemic sclerosis. SNP, single-nucleotide polymorphism; OR, odds ratio; CI, confidence interval. IVW, inverse-variance weighted; IVW(mre), inverse-variance weighted(multiplicative random effects).

Similarly, we explored the effect of CTDs on PBC by using CTDs as exposure and PBC as outcome. Analysis of the results from IVW indicated that there was no evidence to support a causal relationship between CTDs (SLE, RA, SS, and SSc) and PBC (Fig 3), MR-egger method was used to assess pleiotropy, indicated that the presence of horizontal pleiotropy was unlikely to bias the causal relationship between CTDs and RA. Additionally, the “leave-one-out” method ensured the robustness of the results. In conclusion, there is no evidence that genetically predicted CTDs do not lead to an increased risk of PBC.

## 4 Discussion

To the best of our knowledge, this is the first study to validate the causal relationship between PBC and SLE, SS and SSc using MR and large-scale GWAS. Our study reported that PBC increased the risk of SLE, RA, and SS, suggesting an important role of PBC in the pathogenesis of these three CTDs. However, no inverse relationship was observed, i.e., CTDs did not increased increase the risk of PBC. Moreover, PBC did not increase the risk of SSc.

Our study reported that genetically predicted PBC increases the risk of developing SLE. PBC predominantly affects middle-aged and older women, whereas SLE is most often seen in women of childbearing age. Therefore it seems likely that the coexistence of PBC with SLE is low. However, PBC combined with SLE has been reported at a rate of 1.25–3.6% in PBC [14, 17–19] and 1.4–7.5% in SLE [20–22], which seems to indicate the existence of a similar genetic susceptibility to SLE and PBC [23]. Although the pathophysiologic mechanisms underlying the association between PBC and SLE are not fully understood, our study enhanced the understanding by revealing a causal relationship between the two diseases. It is reported that although hepatic dysfunction is the main cause of death in PBC, the cause of death in patients with PBC-SLE is mainly the disease activity or SLE-related complications. The lower survival rate in patients with SLE-PBC is the delayed diagnosis of SLE and more accumulated damage at the baseline [24]. This suggested the importance of early screening of potential SLE among PBC patients with PBC.

The MR results suggest that PBC increases the potential risk of RA. Both RA and PBC are prevalent in middle-aged women. In several large cohort studies, the prevalence of PBC in combination with RA ranged from 1.8% to 13% [11, 14, 18, 25, 26], and RA combined with PBC was also reported [21, 27]. Meanwhile, a genetic study reported that RA and PBC share HLA-DQB1, STAT4, IRF5, MMEL1 and CTLA4 genes [28]. A recent study exploring the genetic link between RA and three autoimmune liver diseases found that PBC increased the risk of RA, and our study found the same result. This suggested that serological antibodies to RA should be screened in patients with PBC with arthralgia and presence of synovitis should be monitored in the ultrasound of joints. If synovitis is detected in a PBC patient with arthralgia, early intervention is essential.

The MR analysis reported that PBC increases the risk of SS. SS is the CTD that is most commonly associated with PBC, with a prevalence of 10%-66%[6, 9, 17, 29]. It has been suggested that these two diseases exhibit a similar immune response in the face of infection, exhibiting inflammation the infiltration of CD4+ T-cells under cytophagy by the antigen IGAs [30]. Moreover, they share similar symptoms, e.g., fatigue and itching, and a similar genetic and pathogenic [30, 31]. A cohort study reported that patients with PBC with comorbid SS were more likely to have fever and an elevated ESR [25]. This indicated that in patients with PBC, antibodies to SS should not be screened focusing on SS-specific manifestations. Glucocorticoids and immunosuppressants are beneficial if PBC is combined with SS.

Our study reported that genetically predicted PBC increases the risk of developing SSc. Several studies have reported PBC combined with SSc [14, 32, 33], It is the first reported CTD in patients with PBC [34]. SSc exhibit similar outcomes as PBC: sclerosis and cirrhosis. A recent study noted that SSc was the most frequent comorbidity in PBC and this rate may still be underestimated [35]. This suggests that we should pay more attention to PBC patients with skin manifestations such as Raynaud’s phenomenon, finger tightness, etc.

Over the past decades, GWAS has successfully identified more than 100 susceptibility loci in the human genome that are closely linked to the HLA locus [36, 37] However, identifying the underlying genetic and environmental factors in autoimmune diseases is not straightforward. Autoimmune disease is a multifactorial disease with multiple influences that can interact with each other [38]. The limitations of observational studies do not allow definitive conclusions to be drawn about the etiology of autoimmune diseases. RCT is the gold standard for assessing causality, however, environmental factors affecting autoimmune diseases are difficult to accurately measure and intervene in. All these issues can be addressed with MR studies of the random distribution of genetic variants during meiosis. In this study, we explored the link between PBC and CTDs with the aim of deepening our understanding of PBC with comorbidity of CTDs. The study can contribute to early diagnosis, accurate stratification, and preemptive treatment, thereby improving the progress of patients.

Our study has some limitations. First, MR must satisfy three major assumptions. Due to the limitation of MR analysis, the second and third assumptions could not be accurately verified. Therefore, we firmly adopted the MR-egger method for horizontal pleiotropy. Second, our study included a European population, which limits the applicability of our findings to other populations. Our study data were derived from several large-scale GWAS, and subgroup analyses were not feasible in the absence of specific demographic data, laboratory indicators, and clinical symptoms. In addition, MR analysis only provided direction for the relationship between PBC and CTD, and the underlying pathological mechanisms should be further investigated.

## 5 Conclusions

To the best of our knowledge, this is the first study to investigated the genetic link between PBC and CTDs and to report that PBC increases the risk of multiple CTDs. Reverse MR indicated that insufficient evidence is available to support that CTDs increase the risk of PBC. To further elucidate the link between PBC and CTD genes, multicenter cohort studies should be conducted in future, using the GWAS database with a larger sample size, and the similar pathogenesis of the two should be explored.

## Supporting information

S1 File (DOCX)

S2 File (DOCX)

S3 File (DOCX)

S4 File (XLSX)

## References

[1] European Association for the Study of the Liver. Electronic address: easloffice@easloffice.eu, European Association for the Study of the Liver. EASL Clinical Practice Guidelines: The diagnosis and management of patients with primary biliary cholangitis. J Hepatol. 2017;67: 145–172. doi: 10.1016/j.jhep.2017.03.022 28427765

[2] Gulamhusein AF, Hirschfield GM. Primary biliary cholangitis: pathogenesis and therapeutic opportunities. Nat Rev Gastroenterol Hepatol. 2020;17: 93–110. doi: 10.1038/s41575-019-0226-7 31819247

[3] Tanaka A. Current understanding of primary biliary cholangitis. Clin Mol Hepatol. 2021;27: 1–21. doi: 10.3350/cmh.2020.0028 33264835 PMC7820210

[4] Bach N, Odin JA. Primary biliary cirrhosis: a Mount Sinai perspective. Mt Sinai J Med. 2003;70: 242–250. 12968197

[5] Shen M, Zhang F, Zhang X. Primary biliary cirrhosis complicated with interstitial lung disease: a prospective study in 178 patients. J Clin Gastroenterol. 2009;43: 676–679. doi: 10.1097/MCG.0b013e31818aa11e 19247207

[6] Liu B, Zhang FC, Zhang ZL, Zhang W, Gao LX. Interstitial lung disease and Sjögren’s syndrome in primary biliary cirrhosis: a causal or casual association? Clin Rheumatol. 2008;27: 1299–1306. doi: 10.1007/s10067-008-0917-x 18512115

[7] Shen M, Zhang F, Zhang X. Pulmonary hypertension in primary biliary cirrhosis: a prospective study in 178 patients. Scand J Gastroenterol. 2009;44: 219–223. doi: 10.1080/00365520802400883 18821172

[8] A case of membranous nephropathy with primary biliary cirrhosis and cyclosporine-induced remission—PubMed. [cited 7 Oct 2023]. Available: https://pubmed.ncbi.nlm.nih.gov/21297326/10.2169/internalmedicine.50.4020 21297326

[9] Deng X, Li J, Hou S, Ci B, Liu B, Xu K. Prevalence and impact of Sjögren’s syndrome in primary biliary cholangitis: a systematic review and meta-analysis. Ann Hepatol. 2022;27: 100746. doi: 10.1016/j.aohep.2022.100746 35970319

[10] Shizuma T. Clinical Characteristics of Concomitant Systemic Lupus Erythematosus and Primary Biliary Cirrhosis: A Literature Review. Journal of Immunology Research. 2015;2015: 1–9. doi: 10.1155/2015/713728 26090497 PMC4452083

[11] Floreani A, Franceschet I, Cazzagon N, Spinazzè A, Buja A, Furlan P, et al. Extrahepatic Autoimmune Conditions Associated with Primary Biliary Cirrhosis. Clinic Rev Allerg Immunol. 2015;48: 192–197. doi: 10.1007/s12016-014-8427-x 24809534

[12] Colapietro F, Lleo A, Generali E. Antimitochondrial Antibodies: from Bench to Bedside. Clin Rev Allergy Immunol. 2022;63: 166–177. doi: 10.1007/s12016-021-08904-y 34586589 PMC8480115

[13] Pollheimer MJ, Fickert P. Animal models in primary biliary cirrhosis and primary sclerosing cholangitis. Clin Rev Allergy Immunol. 2015;48: 207–217. doi: 10.1007/s12016-014-8442-y 25172178

[14] Marasini B. Rheumatic disorders and primary biliary cirrhosis: an appraisal of 170 Italian patients. Annals of the Rheumatic Diseases. 2001;60: 1046–1049. doi: 10.1136/ard.60.11.1046 11602476 PMC1753414

[15] Chalifoux SL, Konyn PG, Choi G, Saab S. Extrahepatic Manifestations of Primary Biliary Cholangitis. Gut Liver. 2017;11: 771–780. doi: 10.5009/gnl16365 28292174 PMC5669592

[16] Emdin CA, Khera AV, Kathiresan S. Mendelian Randomization. JAMA. 2017;318: 1925–1926. doi: 10.1001/jama.2017.17219 29164242

[17] Gershwin ME, Selmi C, Worman HJ, Gold EB, Watnik M, Utts J, et al. Risk factors and comorbidities in primary biliary cirrhosis: A controlled interview-based study of 1032 patients. Hepatology. 2005;42: 1194–1202. doi: 10.1002/hep.20907 16250040 PMC3150736

[18] Watt FE, James OFW, Jones DEJ. Patterns of autoimmunity in primary biliary cirrhosis patients and their families: a population-based cohort study. QJM. 2004;97: 397–406. doi: 10.1093/qjmed/hch078 15208427

[19] Piga M, Vacca A, Porru G, Cauli A, Mathieu A. Liver involvement in systemic lupus erythematosus: incidence, clinical course and outcome of lupus hepatitis. Clin Exp Rheumatol. 2010;28: 504–510. 20609296

[20] Chowdhary VR, Crowson CS, Poterucha JJ, Moder KG. Liver involvement in systemic lupus erythematosus: case review of 40 patients. J Rheumatol. 2008;35: 2159–2164. doi: 10.3899/jrheum.080336 18793002

[21] Takahashi A, Abe K, Yokokawa J, Iwadate H, Kobayashi H, Watanabe H, et al. Clinical features of liver dysfunction in collagen diseases. Hepatol Res. 2010;40: 1092–1097. doi: 10.1111/j.1872-034X.2010.00707.x 20880057

[22] Efe C, Purnak T, Ozaslan E, Ozbalkan Z, Karaaslan Y, Altiparmak E, et al. Autoimmune liver disease in patients with systemic lupus erythematosus: a retrospective analysis of 147 cases. Scand J Gastroenterol. 2011;46: 732–737. doi: 10.3109/00365521.2011.558114 21348808

[23] Carbone M, Lleo A, Sandford RN, Invernizzi P. Implications of genome-wide association studies in novel therapeutics in primary biliary cirrhosis. Eur J Immunol. 2014;44: 945–954. doi: 10.1002/eji.201344270 24481870 PMC4013286

[24] Clinical characteristics and prognosis of concomitant systemic lupus erythematosus and primary biliary cholangitis—PubMed. [cited 8 Oct 2023]. Available: https://pubmed.ncbi.nlm.nih.gov/33067770/10.1007/s10067-020-05457-x 33067770

[25] Wang L, Zhang F-C, Chen H, Zhang X, Xu D, Li Y-Z, et al. Connective tissue diseases in primary biliary cirrhosis: a population-based cohort study. World J Gastroenterol. 2013;19: 5131–5137. doi: 10.3748/wjg.v19.i31.5131 23964148 PMC3746386

[26] Efe C, Torgutalp M, Henriksson I, Alalkim F, Lytvyak E, Trivedi H, et al. Extrahepatic autoimmune diseases in primary biliary cholangitis: Prevalence and significance for clinical presentation and disease outcome. J Gastroenterol Hepatol. 2021;36: 936–942. doi: 10.1111/jgh.15214 32790935

[27] Selmi C, Generali E, Gershwin ME. Rheumatic Manifestations in Autoimmune Liver Disease. Rheum Dis Clin North Am. 2018;44: 65–87. doi: 10.1016/j.rdc.2017.09.008 29149928

[28] Rheumatoid arthritis and primary biliary cirrhosis: cause, consequence, or coincidence?—PubMed. [cited 5 Oct 2023]. Available: https://pubmed.ncbi.nlm.nih.gov/23150824/10.1155/2012/391567 PMC3488395 23150824

[29] Culp KS, Fleming CR, Duffy J, Baldus WP, Dickson ER. Autoimmune associations in primary biliary cirrhosis. Mayo Clin Proc. 1982;57: 365–370. 6896227

[30] Selmi C, Meroni PL, Gershwin ME. Primary biliary cirrhosis and Sjögren’s syndrome: autoimmune epithelitis. J Autoimmun. 2012;39: 34–42. doi: 10.1016/j.jaut.2011.11.005 22178199 PMC3568660

[31] Sun Y, Zhang W, Li B, Zou Z, Selmi C, Gershwin ME. The coexistence of Sjögren’s syndrome and primary biliary cirrhosis: a comprehensive review. Clin Rev Allergy Immunol. 2015;48: 301–315. doi: 10.1007/s12016-015-8471-1 25682089

[32] Ranque B, Mouthon L. Geoepidemiology of systemic sclerosis. Autoimmun Rev. 2010;9: A311–318. doi: 10.1016/j.autrev.2009.11.003 19906362

[33] Rigamonti C, Shand LM, Feudjo M, Bunn CC, Black CM, Denton CP, et al. Clinical features and prognosis of primary biliary cirrhosis associated with systemic sclerosis. Gut. 2006;55: 388–394. doi: 10.1136/gut.2005.075002 16150855 PMC1856066

[34] Sherlock S, Scheuer PJ. The presentation and diagnosis of 100 patients with primary biliary cirrhosis. N Engl J Med. 1973;289: 674–678. doi: 10.1056/NEJM197309272891306 4580473

[35] Norman GL, Bialek A, Encabo S, Butkiewicz B, Wiechowska-Kozlowska A, Brzosko M, et al. Is prevalence of PBC underestimated in patients with systemic sclerosis? Dig Liver Dis. 2009;41: 762–764. doi: 10.1016/j.dld.2009.01.014 19357001

[36] Using GWAS to identify genetic predisposition in hepatic autoimmunity—PubMed. [cited 9 Oct 2023]. Available: https://pubmed.ncbi.nlm.nih.gov/26347073/10.1016/j.jaut.2015.08.016 26347073

[37] Eyre S, Orozco G, Worthington J. The genetics revolution in rheumatology: large scale genomic arrays and genetic mapping. Nat Rev Rheumatol. 2017;13: 421–432. doi: 10.1038/nrrheum.2017.80 28569263

[38] Chen C, Wang P, Zhang R-D, Fang Y, Jiang L-Q, Fang X, et al. Mendelian randomization as a tool to gain insights into the mosaic causes of autoimmune diseases. Autoimmun Rev. 2022;21: 103210. doi: 10.1016/j.autrev.2022.103210 36273526

[39] Cordell HJ, Fryett JJ, Ueno K, Darlay R, Aiba Y, Hitomi Y, et al. An international genome-wide meta-analysis of primary biliary cholangitis: Novel risk loci and candidate drugs. J Hepatol. 2021;75: 572–581. doi: 10.1016/j.jhep.2021.04.055 34033851 PMC8811537

[40] Bentham J, Morris DL, Graham DSC, Pinder CL, Tombleson P, Behrens TW, et al. Genetic association analyses implicate aberrant regulation of innate and adaptive immunity genes in the pathogenesis of systemic lupus erythematosus. Nat Genet. 2015;47: 1457–1464. doi: 10.1038/ng.3434 26502338 PMC4668589

