# Supplementary figures and images for: Genetic link between primary biliary cholangitis and connective tissue diseases in European populations: A two-sample Mendelian randomization study
Source: PLoS One. 2024 Feb 9;19(2):e0298225. doi: 10.1371/journal.pone.0298225 (PMC10857725; doi:10.1371/journal.pone.0298225)

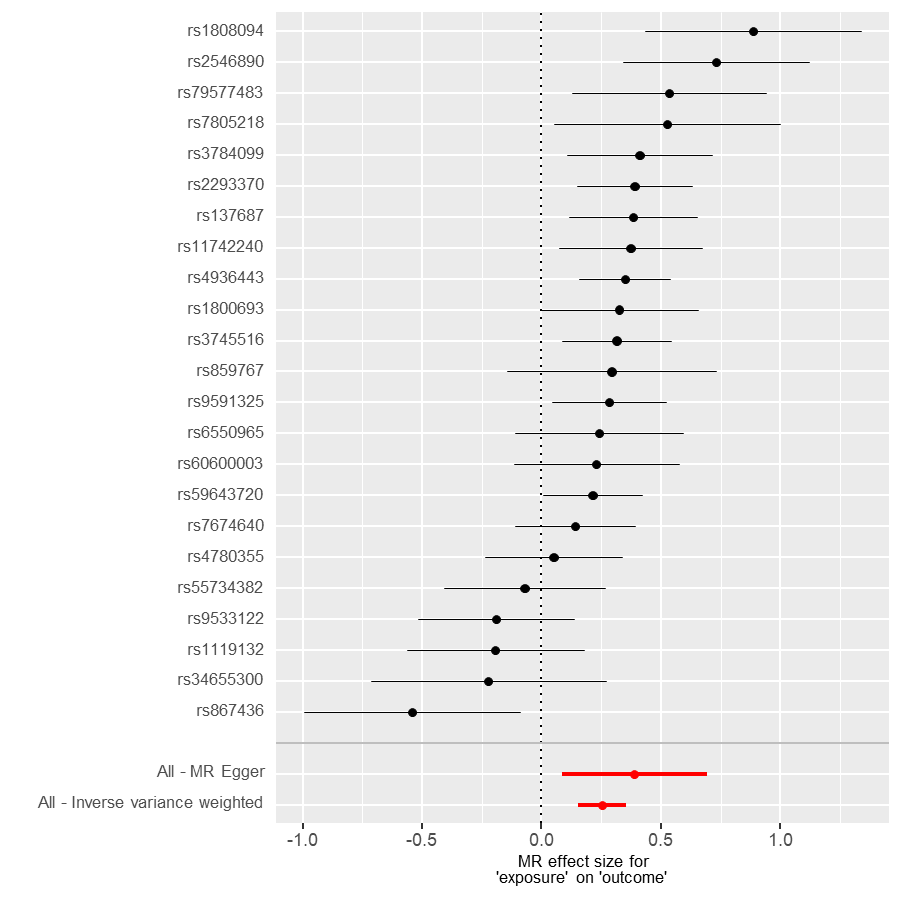


**PBC&SLE
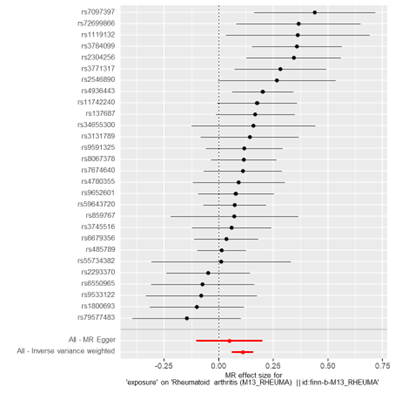
**

**PBC&RA**

**
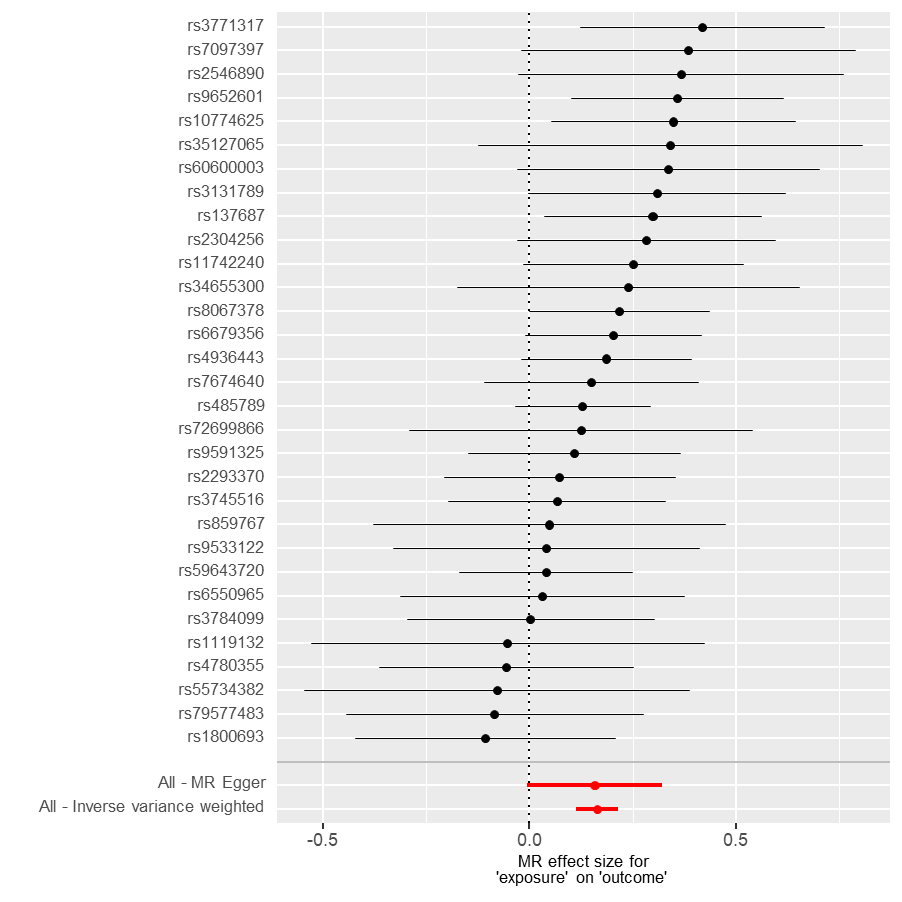
**

**PBC&SS**

**
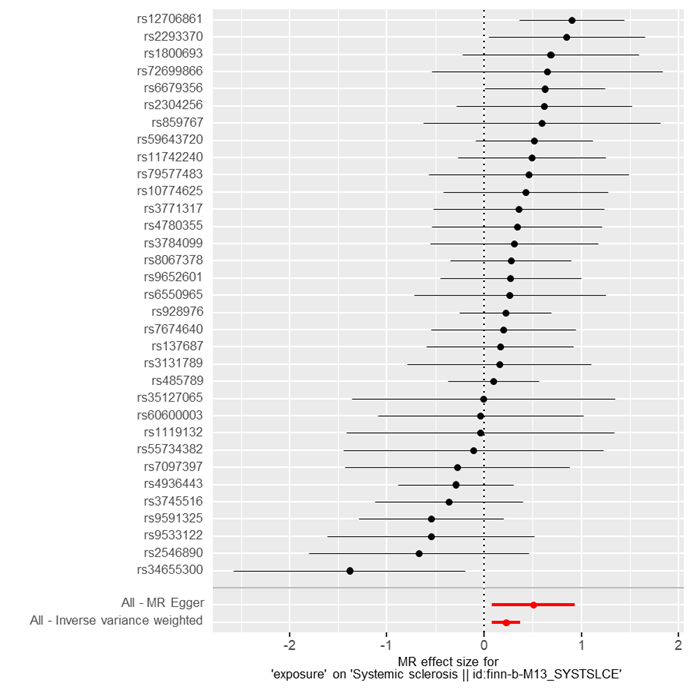
**

**PBC&SSc**

**
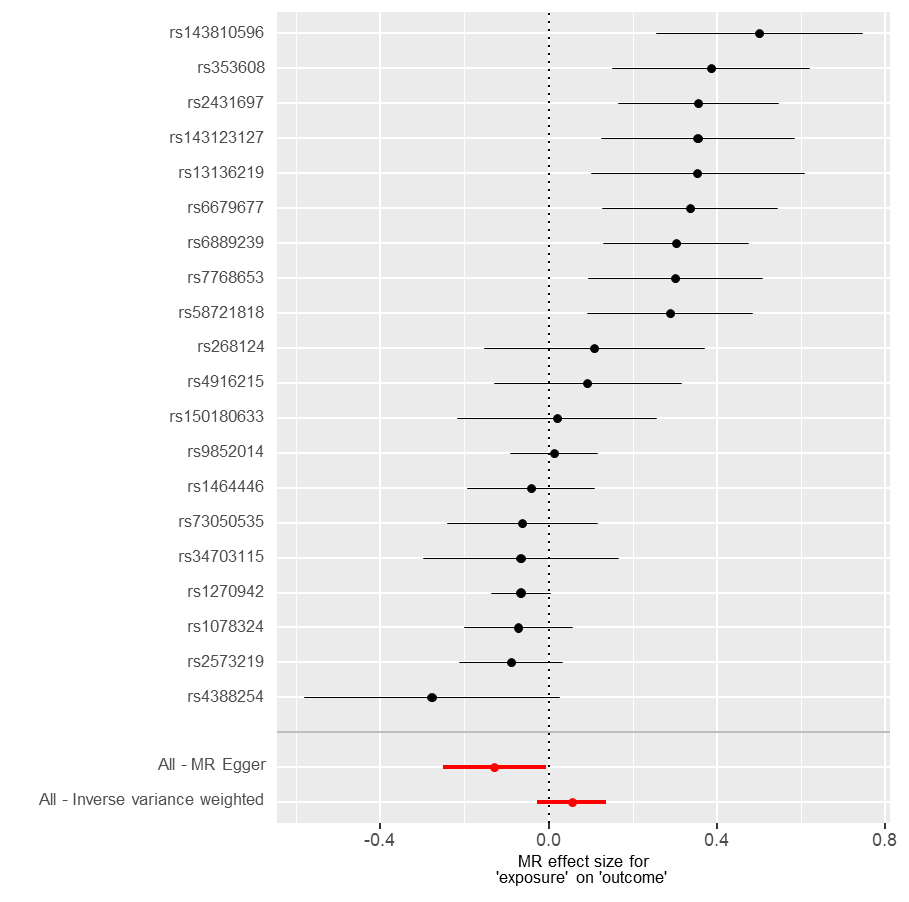
**

**SLE&PBC**

**
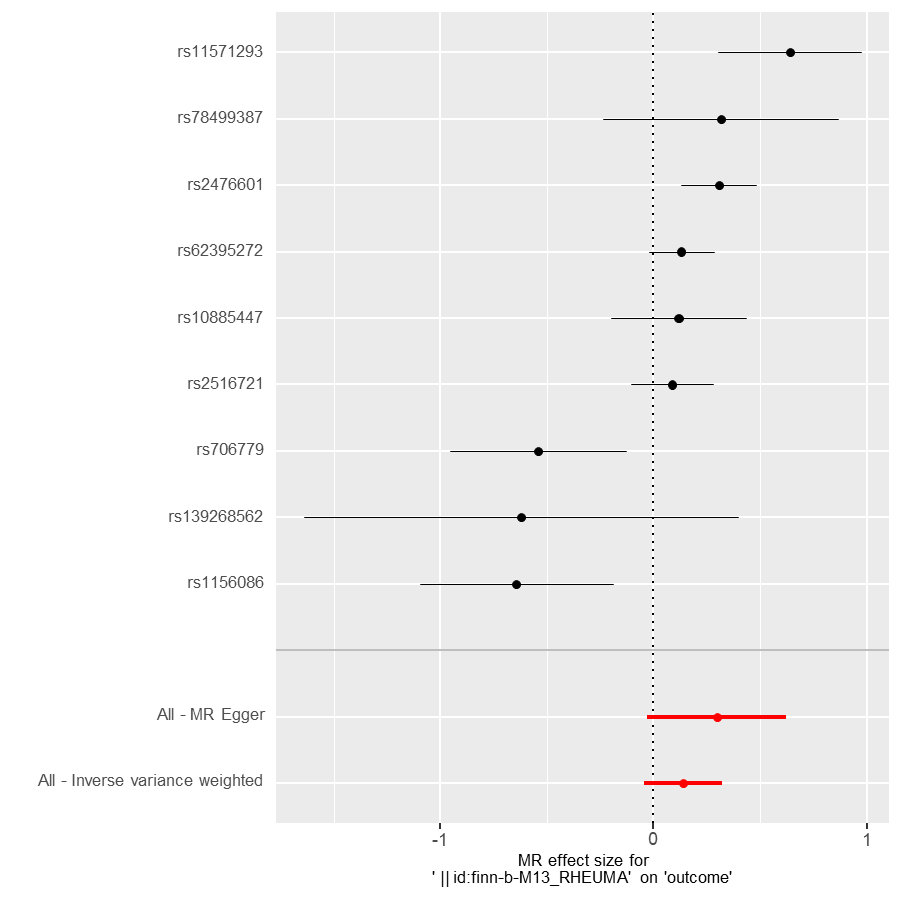
**

**RA&PBC**

**
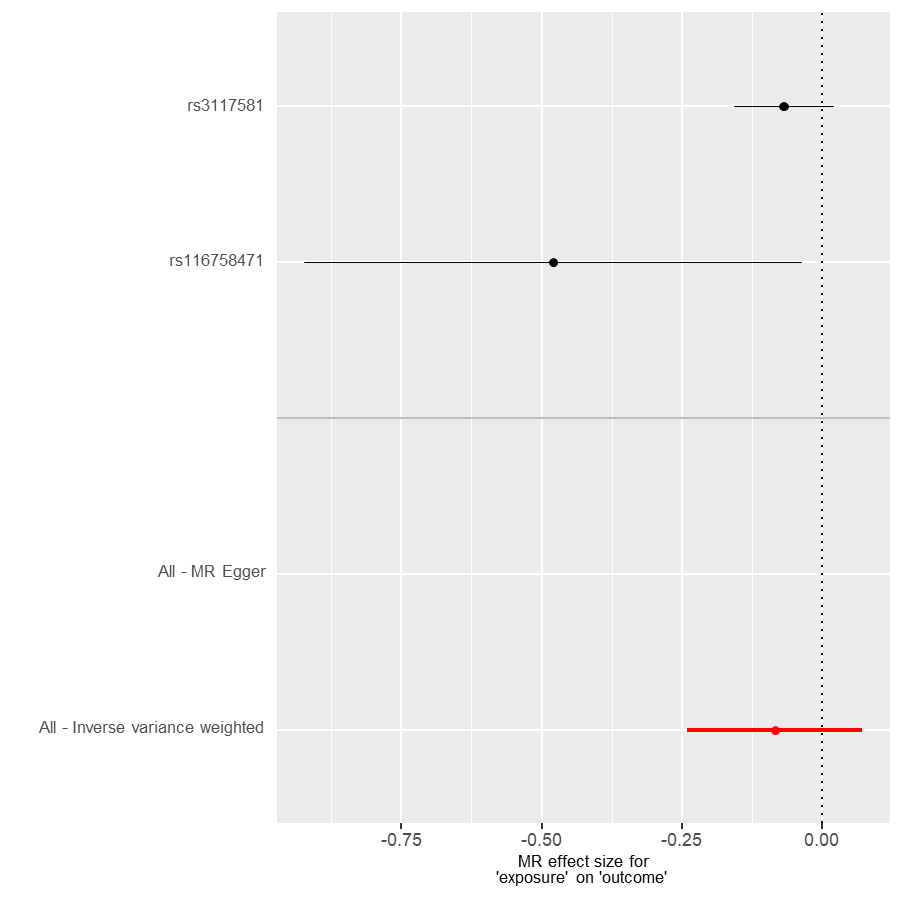
**

**SS&PBC**

**
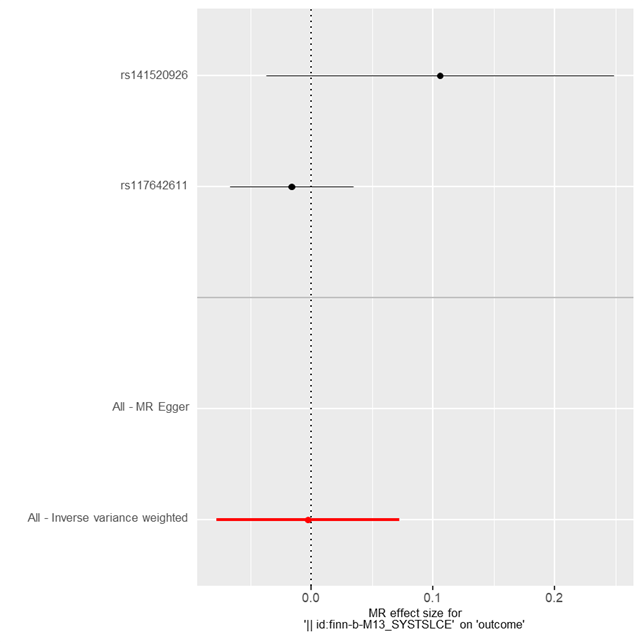
**

**SSc&PBC**

Supplement: S1 File — (DOCX) [file pone.0298225.s001.docx]

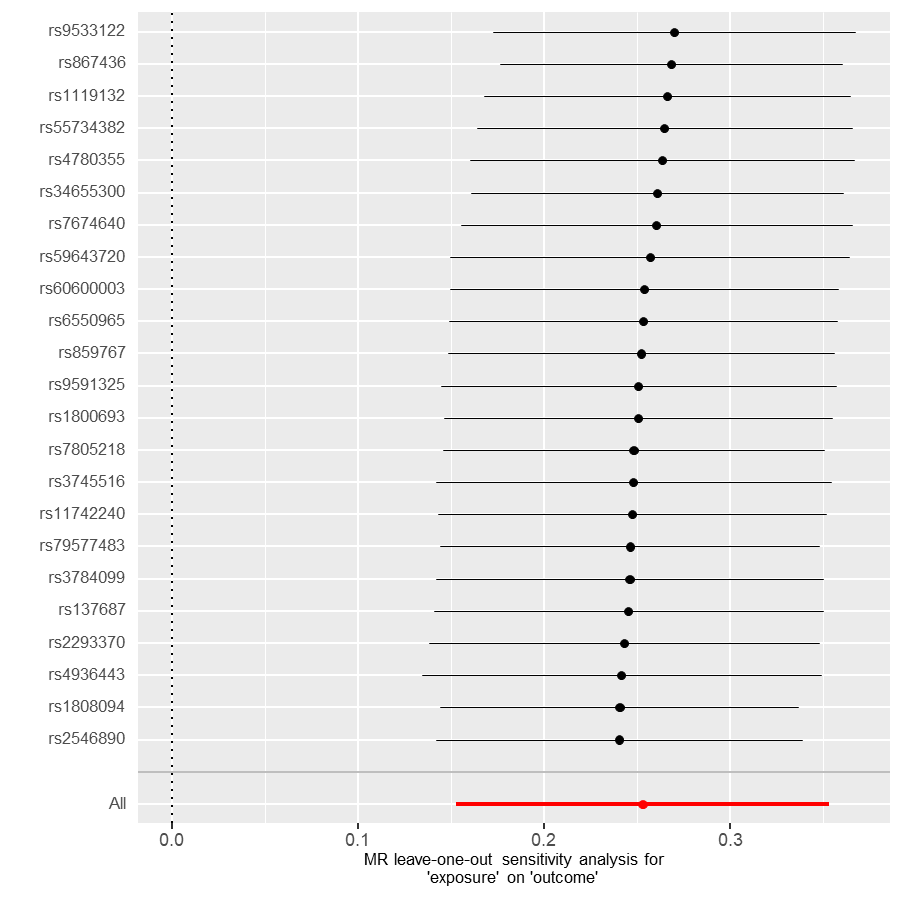

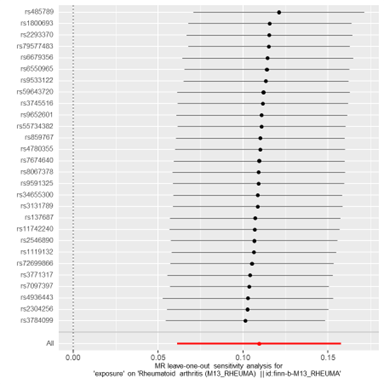


**PBC&SLE PBC&RA**

**
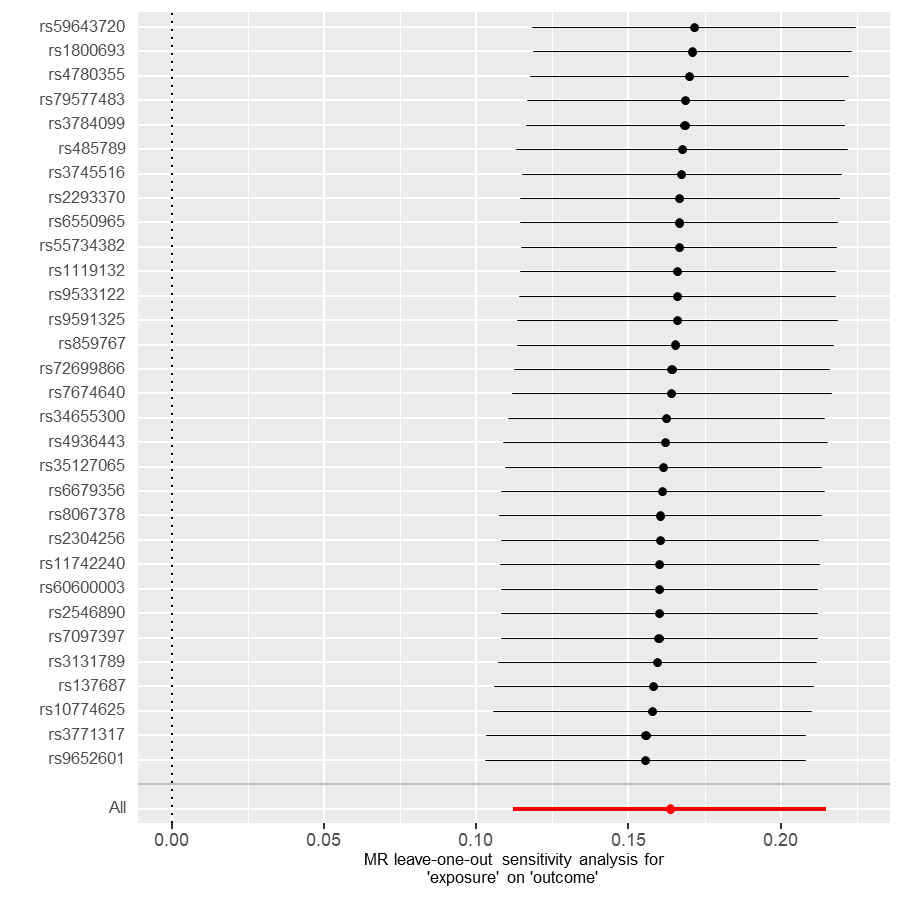

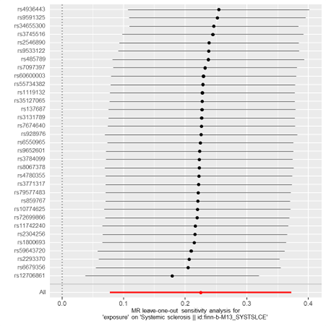
**

**PBC&SS PBC&SSc**

**
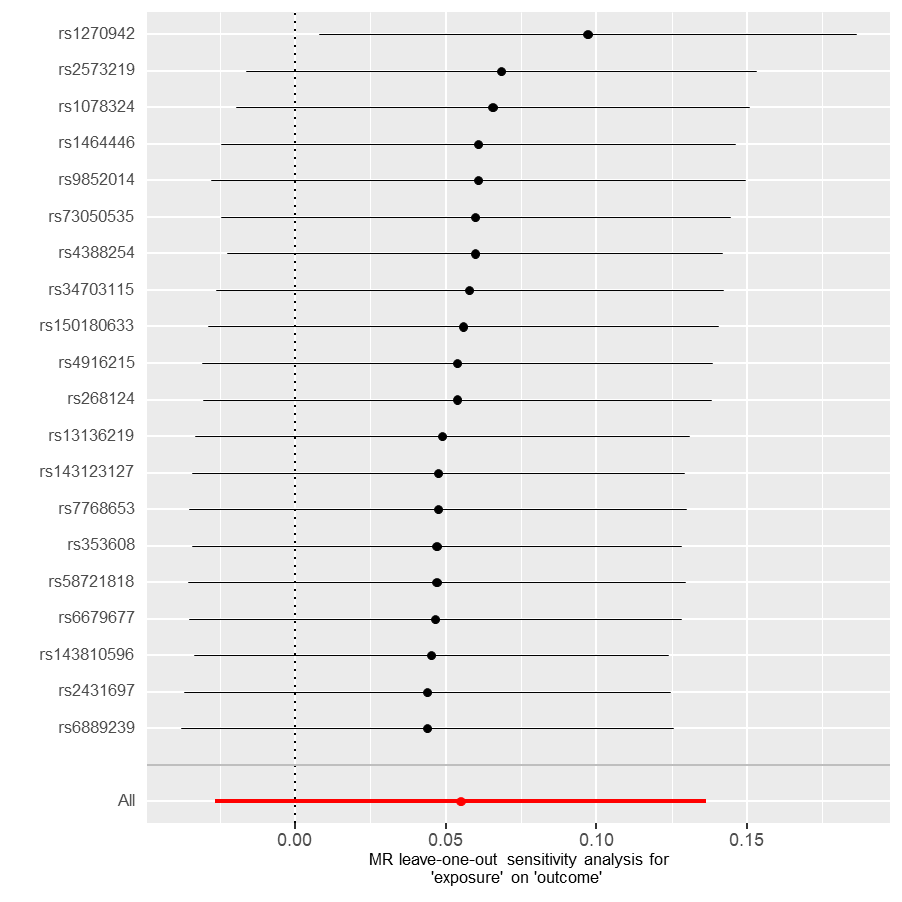

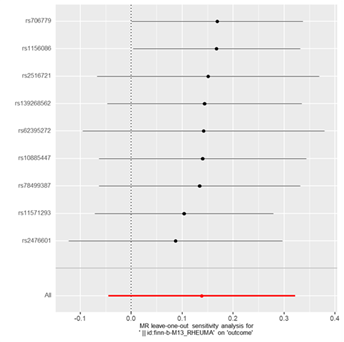
**

**SLE&PBC RA&PBC**

**
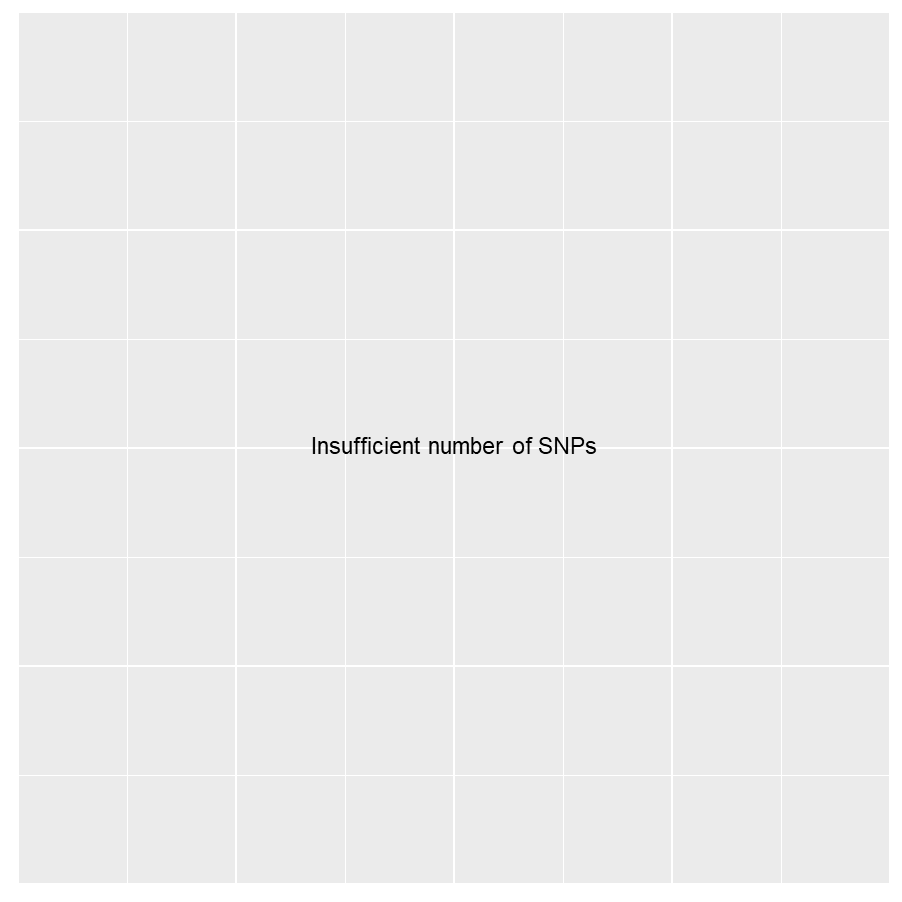

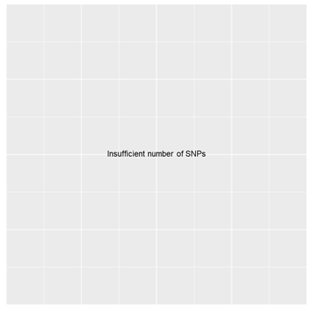
**

**SS&PBC SSc&PBC**

Supplement: S2 File — (DOCX) [file pone.0298225.s002.docx]

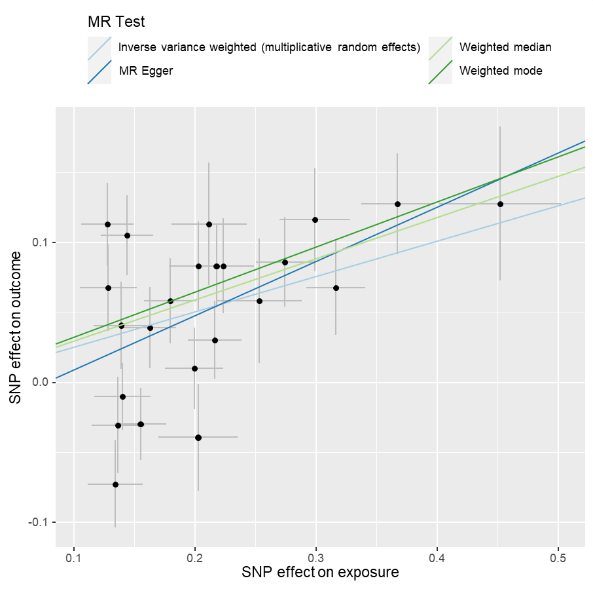

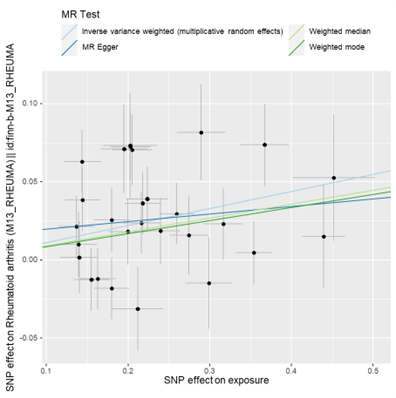


PBC&SLE PBC&RA


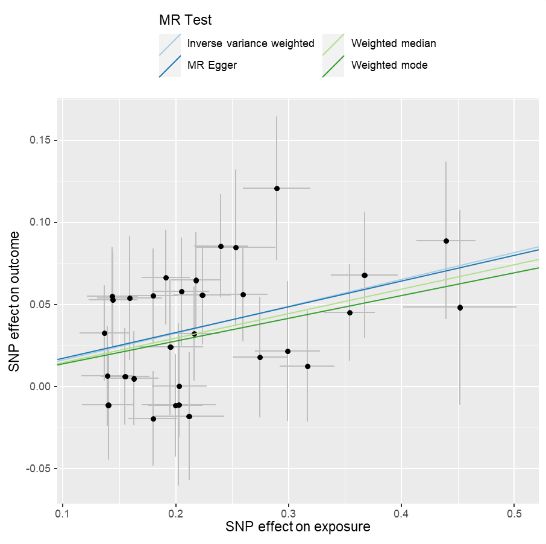

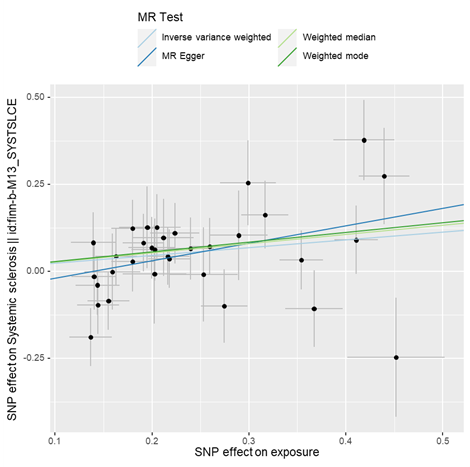


PBC&SS PBC&SSc


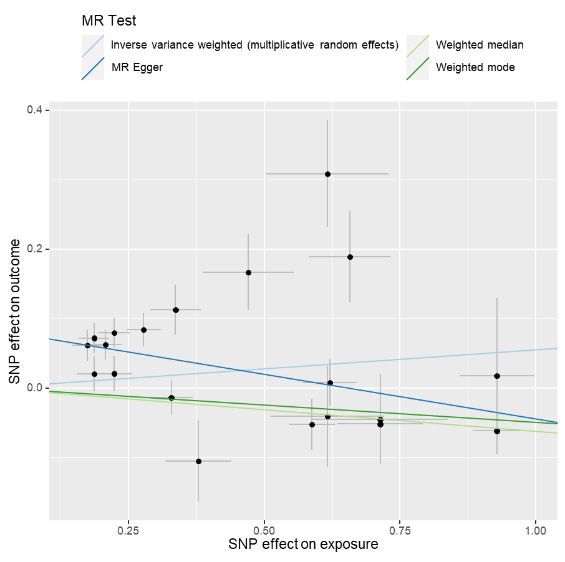

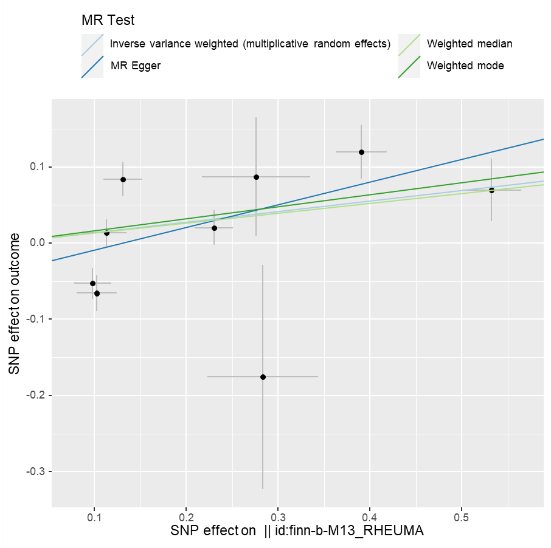


SLE&PBC RA&PBC


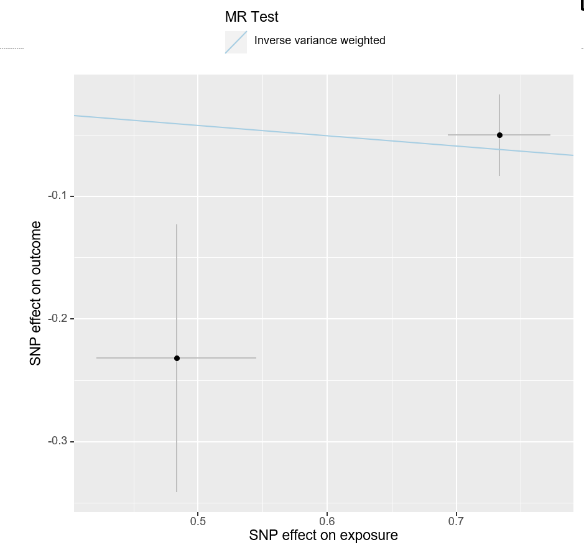

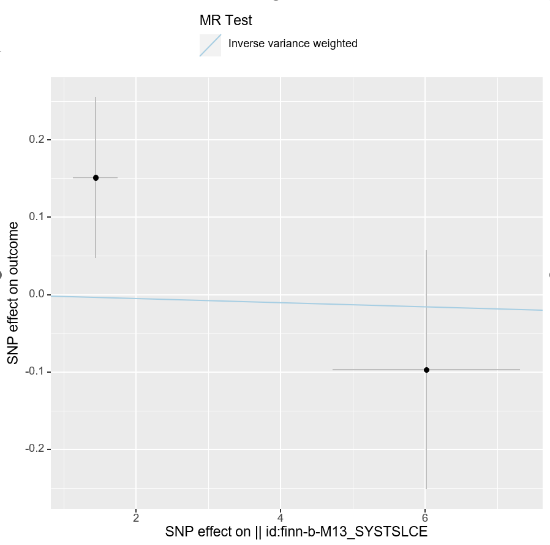


SS&PBC SSc&PBC


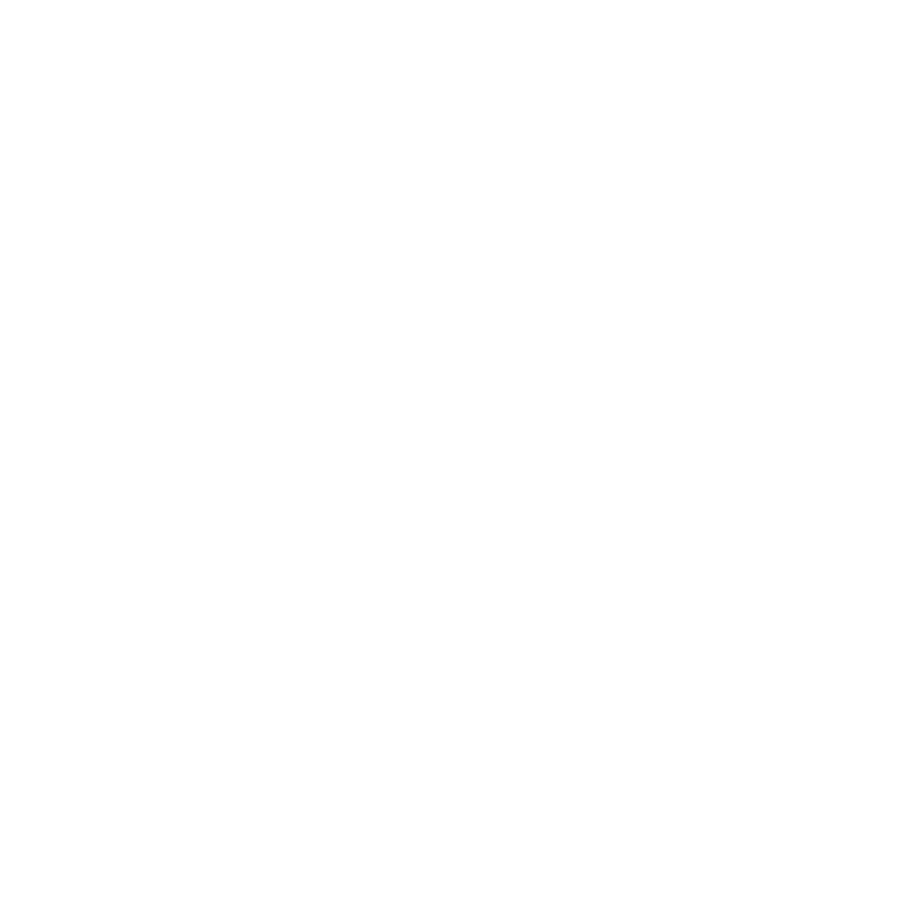

Supplement: S3 File — (DOCX) [file pone.0298225.s003.docx]
